# Chemotherapy and the somatic mutation burden of sperm

**DOI:** 10.1172/jci.insight.188175

**Published:** 2025-05-13

**Authors:** Shany Picciotto, Camilo Arenas-Gallo, Amos Toren, Ruty Mehrian-Shai, Bryan Daly, Stephen Rhodes, Megan Prunty, Ruolin Liu, Anyull Bohorquez, Marta Grońska-Pęski, Shana Melanaphy, Pamela Callum, Emilie Lassen, Anne-Bine Skytte, Rebecca C. Obeng, Christopher Barbieri, Molly Gallogly, Brenda Cooper, Katherine Daunov, Lydia Beard, Koen van Besien, Joshua Halpern, Quintin Pan, Gilad D. Evrony, Viktor A. Adalsteinsson, Jonathan E. Shoag

**Affiliations:** 1 Department of Urology, University Hospitals Cleveland Medical Center, Case Western Reserve University School of Medicine, Cleveland, Ohio, USA.; 2 Pediatric Hemato-Oncology, Sheba Medical Center, Ramat Gan, Israel.; 3 Faculty of Medicine, Tel Aviv University, Tel Aviv, Israel.; 4 Broad Institute of MIT and Harvard, Cambridge, Massachusetts, USA.; 5 Center for Human Genetics and Genomics, New York University Grossman School of Medicine, New York, New York, USA.; 6 California Cryobank, Los Angeles, California, USA.; 7 Tandem Genetics, Los Angeles, California, USA.; 8 Cryos International, Aarhus, Denmark.; 9 Case Comprehensive Cancer Center and; 10 Department of Pathology, University Hospitals Cleveland Medical Center, Case Western Reserve University School of Medicine, Cleveland, Ohio, USA.; 11 Department of Urology, Weill Cornell Medical College, New York, New York, USA.; 12 Division of Hematology and Cell Therapy and; 13 Adult Hematologic Malignancies & Stem Cell Transplantation Service, University Hospitals Seidman Cancer Center, Cleveland, Ohio, USA.; 14 Department of Urology, Northwestern University Feinberg School of Medicine, Chicago, Illinois, USA.; 15 Departments of Pediatrics and Neuroscience & Physiology, New York University Grossman School of Medicine, New York City, New York, USA.

**Keywords:** Genetics, Oncology, Reproductive biology, Cancer, Genetic diseases, Urology

## Abstract

Many chemotherapeutic agents impair cancer growth by inducing DNA damage. The impact of these agents on mutagenesis in normal cells, including sperm, is largely unknown. Here, we applied high-fidelity duplex sequencing to 94 samples from 36 individuals exposed to diverse chemotherapies and 32 controls. We found that in many of the sperm samples from men exposed to chemotherapy, the mutation burden was elevated as compared with controls and the expected burden based on trio studies, with 1 patient having a more than 10-fold increase over that expected for age. Saliva from this same individual also had a markedly higher mutation burden. We then validated this finding using other tissues, also finding an increased mutation burden in the blood and liver of many patients exposed to chemotherapy as compared with unexposed controls. Similarly, mice treated with 3 cycles of cisplatin had an increased mutation burden in sperm but also in the liver and hematopoietic progenitor cells. These results suggest an association between cancer therapies and mutation burden, with implications for counseling patients with cancer considering banking sperm before therapy and for cancer survivors considering the trade-offs of using banked sperm as compared with conceiving naturally.

## Abstract 2

Chemotherapy exposure is associated with an increase in new mutations in sperm in humans and mice.

## Graphical abstract

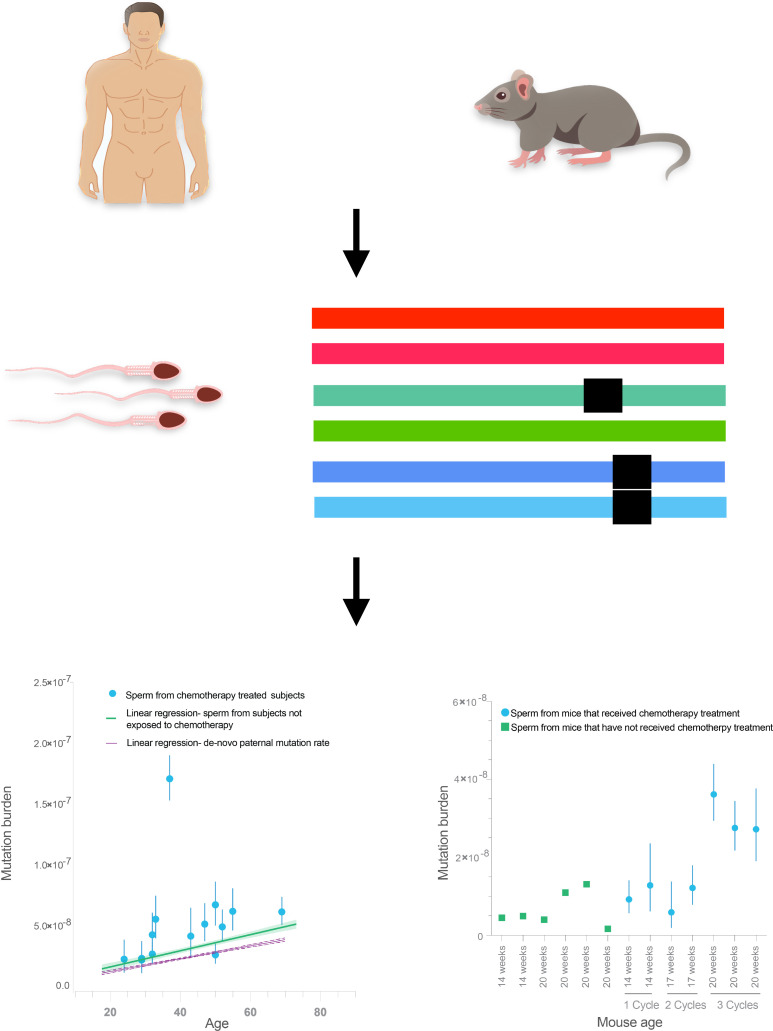

## Introduction

Unlike cancer cells, which begin with a clonal expansion event (1), somatic mutations in healthy cells are generally present at very low mosaicism, either unique to single cells (i.e., unique to single DNA molecules) or present in only small clonal populations, which are below the limits of DNA-sequencing error rates (2). Recent advancements in DNA-sequencing technology have allowed direct measurement of the accumulation of somatic mutations in healthy cells by achieving the fidelity required to detect mutations in single DNA molecules. These studies across diverse tissues have shown that somatic mutations accumulate linearly with age, with different rates and patterns across tissues, reflecting their endogenous and exogenous mutagenic processes (2–10).

When mosaic mutations occur in sperm, they lead to de novo mutations in offspring. Based on sequencing parents and their offspring (trios), it has been shown that paternal age is the main determinant of the number of mutations in offspring, with the vast majority of mutations arising in the paternal rather than maternal germline (11–14). Collectively, new mutations in offspring account for a tremendous burden of disease and have been implicated in diverse conditions, including autism and up to half of severe developmental disorders (13, 15–21).

The mechanism of many cancer therapies is induction of DNA damage in cancer cells. However, the impact of these therapies on mutation rates in normal tissues, including sperm, is poorly defined. Recently, a large study examined 2 cohorts comprising parent-offspring trios from over 21,000 families (22). This study identified 12 offspring with germline hypermutation (up to 6.5-fold normal). Of these 12 outliers, 5 had paternal exposure to chemotherapy before conception, with a platinum treatment signature (SBS31) identifiable in 3 of the 5 individuals. The study further identified 27 fathers who had a history of chemotherapy treatment before conception. Also, 2 out of the 27 fathers had children with a hypermutated genome. This is a substantial enrichment compared with fathers with no history of cancer (22). However, this was limited by a lack of information on the use of banked sperm and details of therapy exposure. This study, the equivalent to sequencing a single sperm and egg from each parent, suggests an effect of chemotherapy on the germline.

Mutagenesis secondary to chemotherapy has important implications for understanding the long-term health effects of cancer therapy in the germline, for counseling patients who desire future fertility. Notably, sperm banking before cancer therapy is often not covered by insurance and is underutilized (23). Further, there are minimal data to guide patients who remain fertile following therapy on the trade-offs of using banked sperm as compared with conceiving naturally. Here, we leverage recent advances in duplex sequencing to directly measure the impact of chemotherapy exposure on mutation burdens in sperm and other tissues.

## Results

### Chemotherapy effect in sperm and saliva.

We collected semen samples from 35 individuals who underwent diverse chemotherapy regimens (Supplemental Table 1; supplemental material available online with this article; https://doi.org/10.1172/jci.insight.188175DS1) at the Department of Urology, University Hospitals Cleveland Medical Center, of whom 14 had adequate sperm in the ejaculate for purification. Specimens were collected between 8 months and 22 years after chemotherapy receipt (Table 1 and Supplemental Table 1). Many of the participants recruited would be considered older fathers; however, both trio and duplex sequencing studies have shown a linear mutation accumulation in sperm with age (3, 24, 25).

After purification (Methods) (26), semen samples were tested for somatic cell contamination using a modification of a previously published method relying on differential methylation at 4 loci (27), which we found was able to detect as little as 1 part blood DNA in 100 parts sperm DNA (Supplemental Figure 1). We identified 1 participant with evidence of somatic contamination and therefore obtained an additional sample from this individual, which was uncontaminated by this assay.

**Figure 1:**
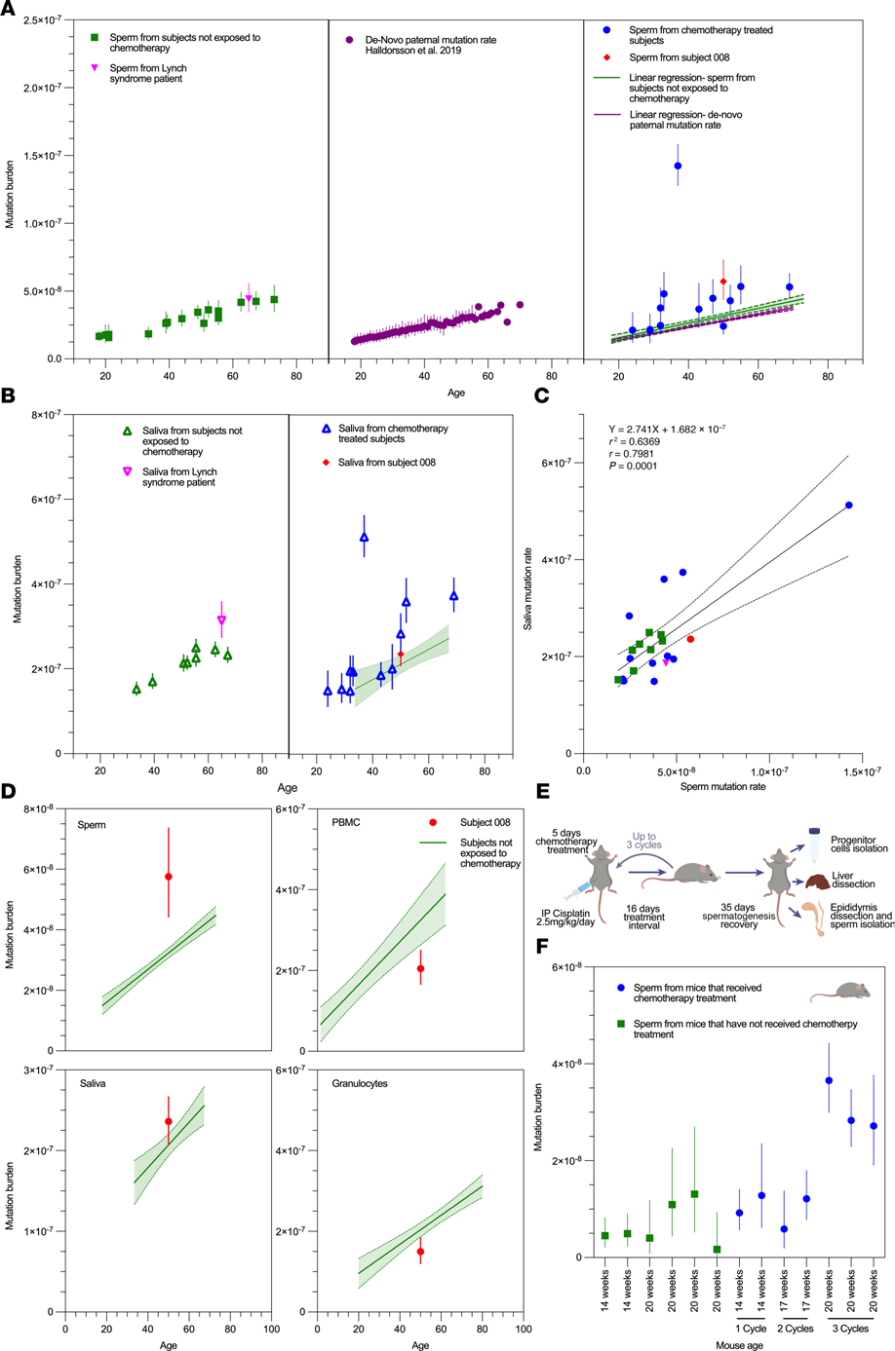
Somatic mutation burden in sperm and saliva in patients exposed to chemotherapy. (**A**) Somatic mutation burden of sperm from patients who received chemotherapy (right panel) as compared with sperm from patients not exposed to chemotherapy (left panel) and the de novo paternal mutation rate (38) (middle panel) from 2,976 whole-genome sequencing trios profiled as part of the Icelandic genealogical database (29). Error bars represent 95%CIs for de novo mutation rates according to paternal age. Linear regression and 95%CI (shaded region) shown for de novo paternal mutation rate and (middle panel) sperm from individuals not exposed to chemotherapy (left panel). (**B**) Saliva mutation rates from the same patients. (**C**) Correlation of sperm and saliva mutation rates (*P* = 0.0001 by Pearson, *r* = 0.7981). (**D**) Somatic mutation rates in sperm, saliva (composed of buccal cells and leukocytes), granulocytes, and PBMCs taken from an individual who underwent chemotherapy and allogenic bone marrow transplant. (**E**) Schematic of experimental design. (**F**) Somatic mutation rate in sperm according to treatment cycle (*P* = 0.003 for 3 cycles versus no therapy compared by Welch’s *t* test). Error bars represent 95% Poisson confidence intervals for estimates.

Using NanoSeq, a recently developed method for high-fidelity duplex DNA sequencing (3), we measured the mutation burden in these specimens as compared with sperm from healthy individuals who did not receive chemotherapy (*n* = 17) (3, 28) and the expected mutation burden in the paternal germline from trio studies generated using microarray and whole-genome sequencing data (Figure 1A) (29). We found that 6 of 14 collected samples had a higher mutation burden than would be expected for the father’s age. Samples from participants treated with chemotherapy had an increase of 9.62 × 10^–9^ (95% confidence interval [95%CI]: 3.48 × 10^–9^ to 1.58 × 10^–8^) in mutation burden, equivalent to an additional 28 mutations in the genome, compared with samples from participants not treated with chemotherapy. Compared with the de novo paternal mutation burden, chemotherapy-treated participants had an increase of 1.30 × 10^–8^ (95%CI: 6.95 × 10^–9^ to 1.90 × 10^–8^) in mutation burden, equivalent to an additional 39 mutations in the genome (Figure 1A). One participant who received chemotherapy had a more than 10-fold increase over the expected burden for age in sperm. When calculating the amount of time it will take to accumulate this amount of mutation based on the linear accumulation rate in sperm (3, 24, 25), it will take around 250 years. This individual received doxorubicin, bleomycin, vinblastine, dacarbazine, ifosfamide, carboplatin, carmustine, etoposide, cytarabine, melphalan, fludarbine, and busulfan 6.5 years before sample collection.

We hypothesized that the interval from the last chemotherapy would not affect the mutation burden, as any double-stranded mutation in stem cells, in theory, would persist. This is concordant with recent data using high-resolution phylogenetic trees of somatic cells (30) (Supplemental Figure 2). Consistent with this, we found no correlation between the mutation burden and the interval between chemotherapy treatment and sample collection (*R*^2^ = 0.05).

To further examine the systemic effects of chemotherapy, we measured the mutation burden in saliva (31) from these same participants. We found chemotherapy was associated with an increase of 4.70 × 10^–8^ (95%CI: 1.48 × 10^–8^ to 8.08 × 10^–8^) in mutational burden, an additional 282 mutations compared with samples from patients not treated with chemotherapy (Figure 1B), with mutations in the sperm and saliva being correlated (*R*^2^ = 0.6369, *P* < 0.001) (Figure 1C). Interestingly, the outlier participant with an outlier high burden (6), as well as an additional participant (8), have undergone allogenic stem cell transplant and therefore received additional conditioning chemotherapy. To avoid any potential confounding because of chimerism in these individuals, we utilized bulk saliva sequencing and combined it with the bulk sperm sequencing (for 008 we also combined bulk granulocyte sequencing) as the germline reference for saliva somatic mutations. The bulk sperm sequencing was also used as the germline reference for sperm somatic mutations. We also confirmed the mutations found in the sperm samples were absent in the participant’s saliva.

In addition to sperm and saliva, we were able to obtain a blood sample from 1 hematopoietic stem cell transplant (HSCT) patient, which we separated into granulocyte and peripheral blood mononuclear cell (PBMC) fractions. Using granulocytes as the germline reference, we found the mutation burden in tissues exposed to chemotherapy in this individual (sperm and a portion of cells in saliva) was higher than the expected mutational burden of non-therapy-exposed participants, the sperm being approximately 2-fold higher. In contrast, when examining the mutational burden of the donor-derived granulocytes and PBMCs, we observed that the mutation burden was lower than expected for the host’s age (the age of the donor is unknown). These findings are consistent with the host, but not donor, cells having been exposed to chemotherapy.

Given the often years-long delay in the recovery of spermatogenesis following therapy, the above specimens were obtained after therapy only (32). We recruited 2 individuals (pre- and posttherapy) and collected specimens 6 months following chemotherapy administration. However, they remained azospermic after therapy. An alternative hypothesis for elevated mutation rates is, therefore, that individuals predisposed to cancer may have higher baseline rates. However, the increase in mutation frequencies in the outlier participant profiled, who does not have a known germline cancer predisposition syndrome, was higher than that reported due to constitutional mismatch repair deficiency (up to 5-fold in blood) (28), polymerase proofreading-associated polyposis (33) (7-fold higher than normal in intestinal crypts and sperm), and MUTYH-associated polyposis (34) (up to 4-fold in colon crypts), supporting an exogenous exposure as causative. We also included the sperm and saliva of a 65-year-old with known high-grade urothelial cancer due to Lynch syndrome (Figure 1), finding saliva and sperm mutation rates within the normal range for his age. This is consistent with a prior study that showed no increase in somatic mutation rates in normal colon crypts of patients with Lynch syndrome (35).

Given the absence of pre/post human sperm, we then assessed the impact of cisplatin, an alkylating agent commonly used for multiple malignancies, including testicular cancer (the most common cancer in young men), on mutation burden in sperm, progenitor cells, and liver using a mouse model (Figure 1E and Supplemental Tables 5 and 6). Previous reports have suggested that spermatogenesis in mice ceases after 4 cycles of cisplatin treatment (36). We found that following 3 cycles of cisplatin treatment, but not 1 or 2, the mutation burden increased markedly in mouse sperm as compared with untreated animals (3.029 × 10^–8^ as compared with 7.435 × 10^–9^, *P* = 0.003; Figure 1F), an addition of 75 mutations per genome. The chemotherapy-associated burden was an order of magnitude higher than that observed in recent studies of the de novo mutation rate in mice (~5 × 10^–9^ per year), approximately 80% of which are accounted for by paternal age (37, 38). Of note, confidence intervals on sperm estimates for mice treated with placebo or fewer than 3 cycles of chemotherapy were wide, despite high duplex sequencing efficiency, due to the very small number of mutations detected in these samples (~1 mutation detected per billion duplex bases sequenced).

### Chemotherapy effect on PBMC mutation burden in pediatric patients.

One prior study using duplex sequencing found that chemotherapy had an effect on the mutational burden in normal blood cells using a high-fidelity sequencing method termed EcoSeq (39). This study measured the mutation burden in peripheral blood cells of 20 pediatric patients with sarcoma, 10 of whom received chemotherapy, identifying an increase in mutational burden following chemotherapy. EcoSeq uses a very small portion (1/90) of the genome for sequencing, which led to detection of very few mutations (between 1 and 16) per patient. Accounting for Poisson error, which was not done in the study report (Supplemental Figure 3), we found these existing estimates inconclusive regarding any potential effects.

**Figure 2:**
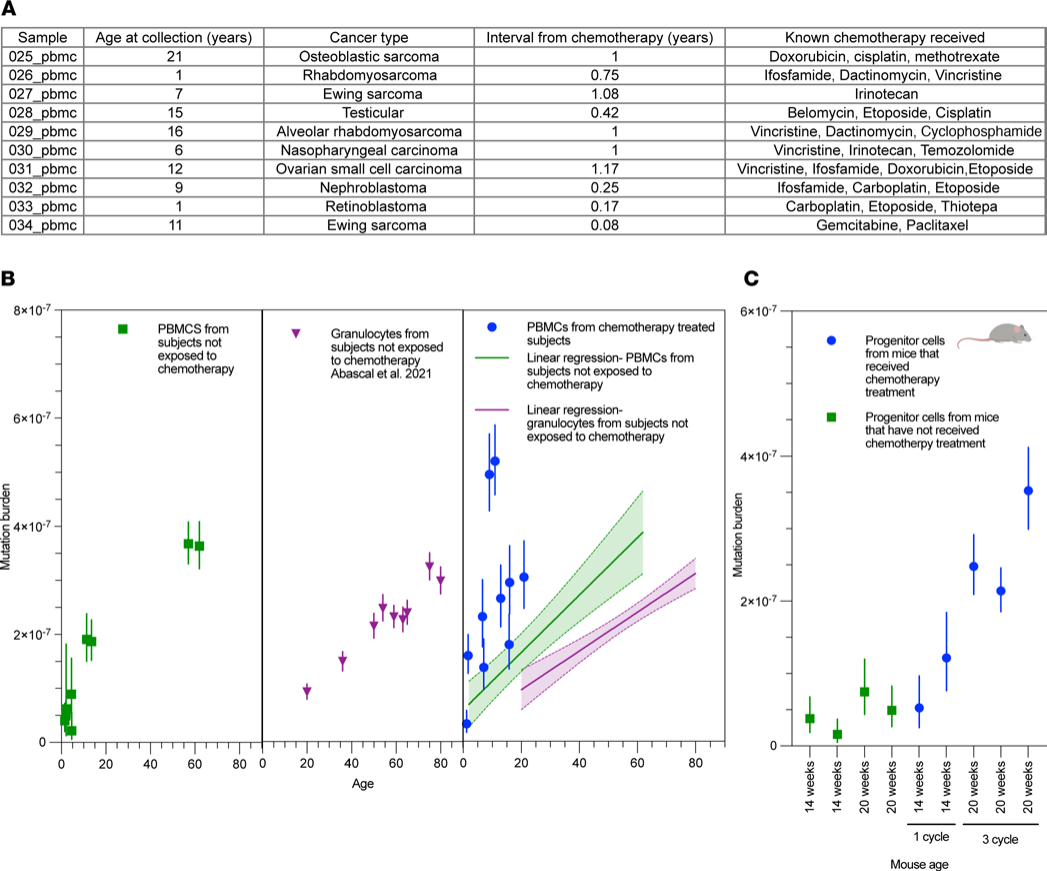
Somatic mutation rate in blood of chemotherapy-exposed patients. (**A**) Age, cancer type, interval from chemotherapy, and regimen of exposed patients. (**B**) Somatic mutation burden of PBMCs from patients who received chemotherapy (right panel) compared with PBMCs from patients not exposed to chemotherapy (left panel) and normal granulocytes profiled in a prior study (3) (middle panel). (**C**) Somatic mutation rate in progenitor cells from mice treated with chemotherapy as compared with those not treated (*P* = 0.03 for 3 cycles versus control compared by Welch’s *t* test). Error bars represent 95% Poisson confidence intervals for estimates.

To assess whether the observed effect in sperm and saliva was consistent in blood, we collected PBMCs from pediatric patients with cancer who had undergone chemotherapy (*n* = 10), patients with cancer who had not undergone chemotherapy (*n* = 7), and healthy controls (*n* = 2) (Figure 2A and Supplemental Table 1). All patients had solid tumors, and chemotherapy regimens included were diverse. Specimens were collected at least 1 month following chemotherapy completion. Chemotherapy was associated with increased somatic mutation burden in PBMCs as compared with patients who did not undergo chemotherapy (Figure 2B), with chemotherapy being associated with an increase of 1.48 × 10^–7^ (95%CI: 4.20 × 10^–8^ to 2.49 × 10^–7^) in mutational burden, an additional 888 mutations per genome. The somatic mutation burden in pediatric patients who received chemotherapy was also substantively higher than that published in granulocytes of adults (Figure 2B) (3). Two patients who received chemotherapy had outlier high mutation burdens. Both patients had completed chemotherapy relatively close to the time of specimen collection (1 month and 3 months), and as such we cannot rule out an effect of cell type as contributing (40, 41). However, the magnitude of effect observed was substantively larger than that attributable to known differences in blood cell type mutation rates, with recent estimates based on single-cell expansion estimating per-cell per-year single nucleotide variant rates of approximately 5 × 10^–9^ in hematopoietic stem cells reaching up to 7.8 × 10^–9^ in memory T cells (40). We then validated these results in progenitor cells from mice. Similar to our findings in sperm, we found that following 3 cycles of cisplatin treatment, the somatic mutation burden increased significantly in mouse progenitor cells as compared with untreated animals (2.714 × 10^–7^ as compared with 6.201 × 10^–8^, *P* = 0.03; Figure 2C), adding 1,424 mutations per genome.

### Chemotherapy and somatic mutation burden in liver tissue.

**Figure 3:**
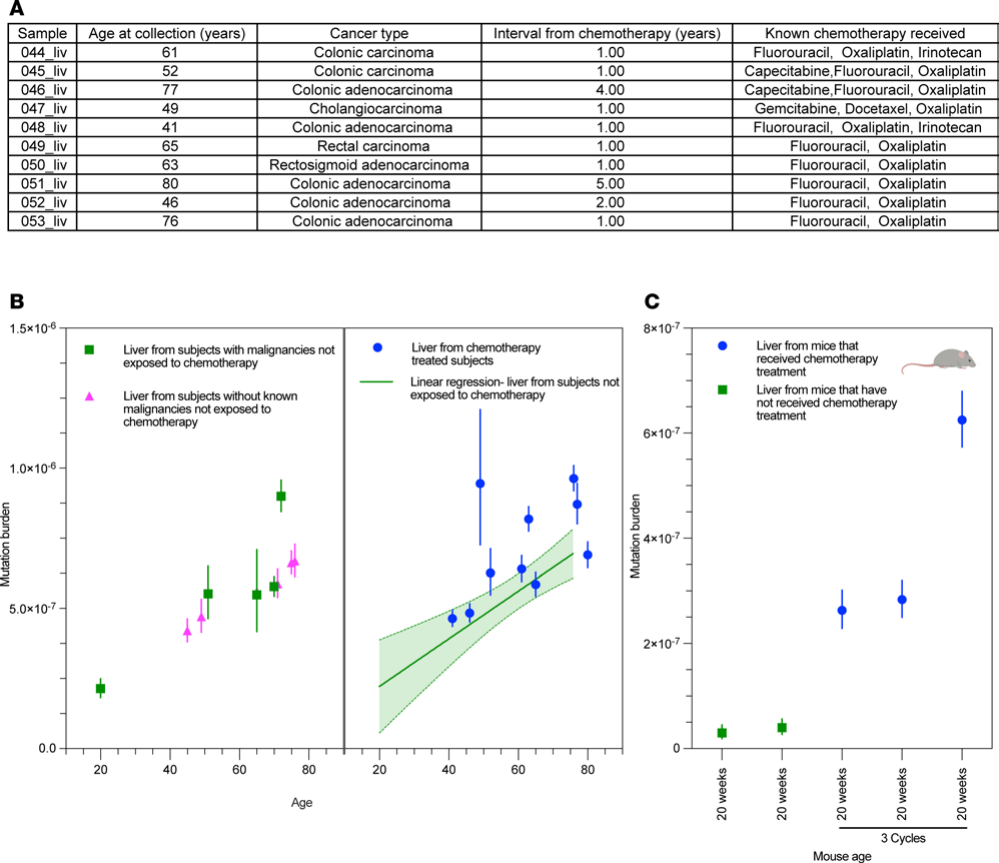
Somatic mutation rate in bulk liver tissue associated with chemotherapy. (**A**) Age, cancer type, interval from chemotherapy, and regimen of exposed patients. (**B**) Somatic mutation burden in liver tissue from patients who received chemotherapy (right panel) compared with liver tissue from patients not exposed to chemotherapy (left panel). (**C**) Somatic mutation rate in liver from mice treated with chemotherapy as compared with those not treated (*P* = 0.009 for 3 cycles versus control compared by Welch’s *t* test, whereas M009_liv is considered an outlier based on a modified *Z*-score of 4.26). Error bars represent 95% Poisson confidence intervals for estimates.

We then sought to evaluate these findings in bulk liver specimens from chemotherapy-exposed individuals, which were chosen based on tissue availability. This included liver tissue from 10 chemotherapy-treated patients as compared with that of patients with cancers who had not received chemotherapy (*n* = 5) and autopsy specimens from individuals without known malignancy (*n* = 6) (Figure 3A). As above, this included patients with diverse malignancies exposed to a range of chemotherapies (Supplemental Table 1). All tissues were reviewed by a trained pathologist to exclude the presence of cancer in the specimen profiled (Supplemental Table 2).

In the liver tissue (>60% hepatocytes) (42), we observed an increase in the somatic mutation burden in patients treated with chemotherapy of 1.20 × 10^–7^ (95%CI: 1.40 × 10^–8^ to 2.32 × 10^–7^), equivalent to an additional 720 mutations (Figure 3B). Our calculated somatic mutation burden in normal, chemotherapy-unexposed liver was consistent with the somatic mutation burden previously published using a clonal expansion technique (7) (Supplemental Figure 4). One patient who had not received chemotherapy was also found to have a relatively high somatic mutation burden (9.00 × 10^–7^, 95%CI: 8.45 × 10^–7^ to 9.58 × 10^–7^). This individual had a history of alcohol abuse, with fibrosis evident on pathologic review. This elevated mutation burden is consistent with previously published data using whole-genome sequencing from microdissections of hepatocytes, which found cirrhosis increases somatic mutation burdens by approximately 4 × 10^–7^ on average (9). Bulk liver tissue from chemotherapy-treated mice also exhibited markedly higher somatic mutation burdens as compared with liver from unexposed mice (3.905 × 10^–7^ as compared with 3.49 × 10^–8^, *P* = 0.009; Figure 3C), adding 2,418 mutations per genome.

### Mutational signature analysis.

Mutational signature analysis identified SBS31, which is associated with cisplatin treatment (43), in 7 samples using a multiple linear regression model (Supplemental Figure 5A). Two of these patients were treated with cisplatin: 1 patient from whom we collected PBMCs (28) and 1 patient from whom we collected saliva and sperm (3). SBS31 was also present in a patient who received etoposide, vincristine, and doxorubicin (14); another who received vincristine, ifosfamide, doxorubicin, and etoposide (31); and another who received cyclophosphamide, doxorubicin, vincristine, and epirubicin (25). We also identified a low proportion of SBS31 signatures explaining 9% of mutations in 1 liver sample not exposed to chemotherapy in an individual on dialysis (57) and in 1 saliva sample from an individual who did not receive chemotherapy (18), explaining 10% of mutations in this specimen. Further, we identified SBS35, which is also associated with platinum chemotherapy (43), in 1 liver sample from a patient exposed to oxaliplatin. Both patients who underwent HSCT had a high proportion of mutations explained by SBS87 in sperm (67% and 92%, Supplemental Table 7), which is associated with thiopurine chemotherapy, though this signature was also identified in many normal samples, perhaps due to overlap with the aging-associated SBS1. Reasons for the absence of signatures detected likely include the small number of mutations detected per sample (particularly in sperm), the multiple therapies patients received, and the diverse origins of Catalogue Of Somatic Mutations In Cancer (COSMIC) mutational signatures (44). An additional signature fitting and extraction method based on flexible Bayesian inference was used (Supplemental Figure 5B and Supplemental Table 8), which did not detect platinum-based chemotherapy signatures.

## Discussion

Here, we find that chemotherapy is associated with an increased mutation burden in sperm that is higher than would be expected for the patient’s age in many individuals profiled. Given the timeline of recovery of spermatogenesis and chemotherapy administration, we were not able to measure pre- and post-therapy specimens, potentially leaving germline predisposition to somatic mutation as a confounder. However, our experiments in mice show an increase in mutation burden following cisplatin in the sperm, liver, and progenitor cells. The known impact of germline alterations suggests therapy as causative. Our results are also consistent with those from a prior large trio study, which found outlier high mutation burdens in the offspring of men exposed to chemotherapy (22). Studies from Swedish national registries have suggested that paternal chemotherapy receipt was not associated with an increase of congenital malformations in testicular cancer survivors (45, 46). However, these studies were limited by an absence of information on the use of banked sperm, and offspring were not evaluated for many conditions linked to de novo mutations. As noted above, despite being elevated in many of the participants, mutation burdens were highly variable among chemotherapy-exposed participants across tissues, with no effects observed in the kidney in the small number of specimens analyzed (Supplemental Figure 6). This variability may be secondary to the differences in pharmacogenomics, the mutagenic potential of the regimens received, the permeability of the blood testis barrier (for sperm), and differences in DNA repair between tissues and individuals.

Our observed increase in the mutation burden of PBMCs in pediatric patients is consistent with the results of a recent study (47), which found an increase in mutational burden in hematopoietic stem and progenitor cells in 24 pediatric patients with cancer (most with hematologic malignancies), as well as the results of Ueda et al. (39). Our results in the liver are also consistent with a recent study, which measured somatic mutations by organoid expansion of stem cells from the colons and livers of 14 patients with cancer (48). In light of emerging evidence that somatic mutation is a biomarker for, if not a cause of, aging, these data suggest that some of the known long-term effects of chemotherapy, such as an increased prevalence of secondary malignancies and perhaps aging-related diseases (49), may be related to therapy-induced somatic mutation.

When analyzing mutational signatures, we used 2 approaches: multiple linear regression and flexible Bayesian inference. With the first approach, we were able to detect signatures SBS31 and SBS35, which are associated with platinum treatment in a few samples, but were not able to detect these signatures using flexible Bayesian inference. Difficulty in fitting platinum signatures in sperm is almost certainly due to the low number of mutations detected per sample (50), and lower frequency signatures can be miscalled or mixed with other signatures, making them very difficult to detect (51).

In conclusion, we show here by high-fidelity duplex sequencing that a substantial proportion of individuals with a history of chemotherapy exposure had an increase in the mutation burden in sperm as well as other tissues. While larger scale studies are needed to determine which therapies are most likely to induce somatic mutations or develop clinical tests to directly measure somatic mutation rates in individual patients, we would suggest these results support the potential value of banking sperm prior to cancer therapy. Further, they preliminarily suggest that the use of sperm banked before therapy, when available, may be preferable to natural conception for cancer survivors who remain fertile following chemotherapy, to minimize the small, but real, risks associated with an increased burden of paternal de novo mutations.

## Methods

### Sex as a biological variable.

In human liver and PBMC samples, sex was not considered as a biological variable. For in vivo studies, only male mice were used in order to study the effect of chemotherapy on sperm as well as their other tissues.

### DNA extraction from sperm samples.

Semen samples were processed within 1 hour after procurement at the laboratory. Samples were subjected to liquefaction in sperm-washing media for 15 minutes at 37°C. The liquefied semen was washed with preheated sperm-washing medium (Irvine Scientific) and centrifuged at 300*g* for 5 minutes. The pellet was resuspended in 1.5 mL sperm-washing medium and added carefully above the PureSperm 40/80 sperm gradient (Nidacon) for sperm isolation. The sample was then centrifuged at 300*g* for 20 minutes. The pellet was resuspended in 250 μL PBS and examined under a microscope to ensure the presence of only motile sperm cells. RLT lysis buffer (QIAGEN) containing 2.5 mM TCEP (Thermo Fisher Scientific) was added to the sample, and lysis was performed by adding 0.2 mm sterile stainless steel beads and shaking the sample in a BeadBug microtube homogenizer (Benchmark Scientific) for 4 minutes at maximum speed. After lysis, DNA was extracted using a QIAamp DNA Mini Kit (QIAGEN). Briefly, AL buffer (part of the QIAamp DNA Mini Kit), followed by ethanol, was added to the lysate and vortexed. The lysate was loaded onto a QIAamp DNA mini spin column and washed with AW1 and AW2 washing buffers. DNA was eluted in 100 μL of 10 mM Tris pH 8. The extracted DNA was treated with Monarch RNase A (New England BioLabs; NEB) for 5 minutes and cleaned using SPRIselect beads (Beckman Coulter). We added 0.8× beads to the DNA samples and incubated for 7 minutes. The supernatant was removed following the placement of the tube on a magnet. The bead pellet was washed twice with 80% ethanol, left to dry, and then resuspended in 35 μL nuclease-free water. Following 7 minutes of incubation, the supernatant containing the DNA was collected. Photos of the sperm utilized are included in Supplemental Figure 7.

### Mouse cisplatin treatment.

Male 8-week-old C57BL6/J mice purchased from The Jackson Laboratory with a mean weight of 25 g were treated with cisplatin at 2.5 mg/kg/d of cisplatin in 200 μL of 0.9% saline (Sigma-Aldrich). For sperm analysis, a placebo group was administered the identical volume of 0.9% saline without cisplatin, while for the liver comparator group, no treatment was administered. The mice underwent up to 3 cycles of treatment, each cycle consisting of daily intraperitoneal injections for 5 consecutive days, followed by a 16-day recovery phase. Given the length of the spermatogenic cycle in mice is approximately 35 days (36), mice were sacrificed 35 days following the last chemotherapy dose. After the completion of each cycle, 2 mice were euthanized, and tissue was collected.

### Mouse sperm extraction.

Immediately after euthanasia with isoflurane, a medial laparotomy was performed for macroscopic dissection of the testicles. Each mouse had both testes removed along with its ductus deferens. The excised tissues were subsequently transferred to a Petri dish filled with sperm-handling medium at 24°C (Fujifilm 90166). Using microscopic dissection (OLYMPUS SZ51), the epididymis along with ductus deferens were separated from the testis and transferred to a new clean Petri dish containing 3 mL of 24°C sperm-handling medium. The epididymis was then opened, the content was suspended in media, and all residual tissue pieces were discarded.

The resulting mixture of media and sperm was analyzed microscopically at 20× original magnification to assess motility and the presence of sperm cells. Subsequently, 3 mL of media with sperm cells was subjected to centrifugation using a tilt bucket rotor (550 RCF for 5 minutes). The resultant pellet was resuspended in 1 mL of handling medium at 24°C and centrifuged once more (550*g* for 5 minutes) using an 80/40 gradient (PureSperm PSK-020) to remove dead sperm and nonsperm cells, and the resulting pellet was subsequently used for sperm DNA extraction. Photos of the sperm utilized are included in Supplemental Figure 8.

Sperm samples from mice M010 and M011 showed high levels of repetitive mutations and were considered contaminated and excluded from the study. Note the germline sperm libraries from these mice were not contaminated and were used as germline references for these mice.

### Progenitor cell separation from bone marrow.

Two femur bones from each mouse were removed and cleaned. A total of 2 mL PBS was used to wash both femurs to collect bone marrow. Progenitor cells were purified from bone marrow using the EasySep Mouse Hematopoietic Progenitor Cell Isolation Kit (STEMCELL Technologies). Following purification DNA was extracted from the cells using a QIAamp DNA Mini Kit. The cells were washed and resuspended in 200 μL PBS. Proteinase K (20 μL) was added to 200 μL AL buffer. The sample was vortexed and incubated at 56°C for 10 minutes. Ethanol (100%) was added to the lysate, and the mixture was vortexed. The lysate was loaded onto a QIAamp DNA mini spin column and washed with AW1 and AW2 washing buffers. DNA was then eluted in 100 μL nuclease-free water.

### DNA extraction from frozen tissue.

Tissues were lysed, and DNA was extracted using the QIAamp DNA Mini Kit (QIAGEN). Frozen tissue (25 mg, liver or kidney) was cut into small pieces, placed in a 1.5 mL tube with ATL buffer (part of the QIAamp DNA Mini Kit) and proteinase K, and then vortexed. For mouse liver tissue, the left lobe of the liver was harvested. The samples were incubated at 56°C for 2–3 hours until they were completely lysed. The lysate was treated with RNase A for 2 minutes, followed by the addition of AL buffer and incubation at 70°C for 10 minutes. Ethanol (100%) was added to the lysate, and the mixture was vortexed. The lysate was loaded onto a QIAamp DNA mini spin column and washed with AW1 and AW2 washing buffers. DNA was eluted in 200 μL of nuclease-free water.

### DNA extraction from PBMCs.

Blood was collected into EDTA tubes and processed within 2 hours of collection. PBMCs were isolated from whole-blood samples using a Lymphoprep gradient (STEMCELL Technologies). Blood was layered above the gradient and centrifuged at 800*g* for 20 minutes. PBMCs were carefully removed and washed in PBS twice and centrifuged at 400*g* for 10 minutes. After the second wash, the cell pellet was resuspended and stored at –80°C in freezing media (90% FBS 10% DMSO) awaiting DNA extraction.

DNA was extracted from the cells using a QIAamp DNA Mini Kit. The cells were washed and resuspended in 200 μL PBS. Proteinase K (20 μL) was added to 200 μL AL buffer. The sample was vortexed and incubated at 56°C for 10 minutes. Ethanol (100%) was added to the lysate, and the mixture was vortexed. The lysate was loaded onto a QIAamp DNA mini spin column and washed with AW1 and AW2 washing buffers. DNA was then eluted in 100 μL nuclease-free water.

### DNA extraction from saliva.

DNA was extracted from saliva using the QIAamp DNA Mini Kit, according to the manufacturer’s protocol.

### Somatic cell contamination assay.

Sperm purity was assessed for all samples via a previously published assay (27). A total of 350 ng DNA from each sample was bisulfite-treated using the EZ DNA Methylation Kit (Zymo Research). The bisulfite-treated DNA was then amplified by PCR in 4 loci, namely PCR7, PCR11, PCR12, and PCR31, using the primer sets specified below. The loci PCR7, PCR11, and PCR31 are methylated in sperm but not in somatic cells, whereas PCR12 is methylated in somatic cells but not in sperm. Primers were PCR7 (GGGTTATATGATAGTTTATAGGGTTATT and TCTATTACTACCACTTCCTAAATCAA), PCR11 (TGAGATGTTTGTTAGTTTATTATTTTGG and TCATCTTCTCCCACCAAATTTC), PCR12 (TAGAGGGTAGTTTTTAAGAGGG and ATTAACCAACCTCTTCCATATTCTT), and PCR31 (TTTTAGTTTTGGGAGGGGTTGTTT and CTACCAAAATTAAAAACCAACCCAC). PCRs were carried out in a final volume of 20 μL, which contained 1.5 μL of bisulfite-converted DNA, 10 μL of 2× ZymoTaq PreMix (Zymo Research), PCR primers, and nuclease-free water. The final concentrations of each forward and reverse primer were as follows: 0.6 μM for PCR7 primers, 0.6 μM for PCR11 primers, 0.3 μM for PCR12 primers, and 0.45 μM for PCR31 primers. The cycling conditions for the PCRs were as follows: initial denaturation at 95°C for 10 minutes, followed by 40 cycles of denaturation at 94°C for 30 seconds, annealing at X°C for 30 seconds, and extension at 72°C for 30 seconds, and a final extension step at 72°C for 7 minutes. The annealing temperatures (X) were 49°C for PCR7 and PCR11, 51°C for PCR12, and 55°C for PCR31. PCR products were purified using a 2× volumetric ratio SPRI bead cleanup and eluted in 22 μL of 10 mM Tris pH 8. Restriction digestions were performed by combining 5 μL of purified PCR product, restriction enzyme (10 units of HpyCH4IV [NEB] for PCR7 and PCR31, and 20 units of TaqI-v2 [NEB] for PCR11 and PCR12), 1 μL of 10× CutSmart buffer (NEB), and nuclease-free water for a total reaction volume of 10 μL. Restriction digestions were performed at 37°C (HpyCH4IV) or 65°C (TaqI-v2) for 60 minutes. Control reactions without the restriction enzyme were performed for each sample/locus combination. DNA concentration and size were measured using TapeStation (Agilent Technologies).

### NanoSeq library preparation.

DNA libraries for Illumina sequencing were prepared using the protocol for nanorate sequencing (NanoSeq) as previously described (3). DNA was purified using Ampure beads (Beckman Coulter). Fragmentation mix was added to perform on-bead digestion at 37°C for 15 minutes. Ampure bead cleanup was performed, and fragmented DNA was eluted in 10 μL nuclease-free water. A total of 5 μL A-tailing mix was added to the fragmented DNA and incubated at 37°C for 30 minutes. A total of 22.4 μL of ligation mix was added to the A-tail product and incubated at 20°C for 20 minutes. Ampure bead cleaning was performed, and the ligation product was eluted in 50 μL nuclease-free water. A KAPA library quantification kit (Roche) was used to quantify the amount of DNA. The concentration of the library was determined and diluted to the proper femtomole input amount. For sperm, we input 0.6 fmol, and for the liver, kidney, and PBMCs, we input 0.4 fmol. Furthermore, for the filtration of germline SNPs, we sequenced undiluted DNA from the same library, which we refer to as a matched normal. PCR amplification was performed using unique dual indexes containing primers (Paragon Genomics). Two consecutive Ampure bead cleanups were performed, followed by sample quantification and sample pooling.

### Sequencing and data analysis.

The samples were subjected to paired-end (150 bp) whole-genome sequencing at 33% coverage on a NovaSeq platform (Illumina). The sperm samples were sequenced using 300 million reads. The liver, kidney, saliva, and PBMCs were sequenced with 260 million reads. All matched normal samples were sequenced with 100 million reads. Unless otherwise indicated (i.e., for patients who underwent HSCT), sperm was used as the germline reference for both sperm and saliva. To ensure the samples were not contaminated, we ran VerifyBAMID (52) as described in the NanoSeq protocol (3). VerifyBAMID calculates the freemix value, an estimate of contamination using sequence-only method with Brent’s single dimensional optimization (52). Samples with a freemix value of under 0.005 are considered not contaminated. Saliva samples from the patients who underwent HSCT (006_sal and 008_sal) had a high freemix value (Supplemental Table 3), as the saliva samples in these patients contain 2 genomes (patient and donor). 006_sal had a freemix value of 0.24; i.e., 24% or more of nonreference bases are observed in reference sites. Sample 008_sal had a freemix value of 0.22; i.e., 22% or more of nonreference bases are observed in reference sites. To overcome the issue of contamination when calculating mutational burden, the sample was compared with a combined germline-matched normal of the saliva, sperm (6), and granulocyte (8) samples of each participant, including manual review of detected mutations. Sample data were analyzed using the NanoSeq pipeline available at https://github.com/cancerit/NanoSeq (commit ID 4136d3c). The sequences were aligned with the human reference genome (GRCh37, hs37d5 build) using BWA-MEM (https://bio-bwa.sourceforge.net). For mice, the reference mm10 sequence was utilized. Mutational signatures were extracted using the deconstructSigs package for R (53) and SigFit package for R (54). Signatures were fitted to COSMIC (44) v3.2 signatures.

### Statistics.

Somatic mutation rates were modeled as a function of age and group (chemotherapy- or non-chemotherapy-treated) via distributional regression. Age and group were included as additive terms (no interaction). This model allowed for the residual variance to vary by group, to account for the observation that the chemotherapy group exhibits greater variability around the age regression line relative to the nonchemotherapy group. The Poisson 95%CIs associated with the point estimates of the somatic mutation rate were used to calculate standard errors, which were then used to weight observations (analogous to meta-analysis). Group was included in the model as a dummy variable, and the estimates reported in the text represent the shift in intercept of the age regression line for the chemotherapy group relative to the nonchemotherapy group.

Models were fit via Bayesian estimation using the brms (55) package for R (version 4.1.2) (56), which serves as an interface to the MCMC sampling routines in Stan (57). The default prior settings in brms were used (*t* distribution with 3 degrees of freedom on intercept terms and flat prior on other coefficients). Four independent MCMC chains were run for 5,000 samples with the first 2,500 samples discarded as warmup, leaving 10,000 posterior samples. Convergence was assessed via the statistic (58), which was always less than 1.05. The estimates reported in the text are posterior means and 95% credible intervals (the 2.5th and 97.5th percentiles of the posterior samples). A *P* value less than 0.05 was considered significant.

### Study approval.

Fresh frozen liver and kidney tissues were obtained from postmortem specimens from the Human Tissue Procurement Facility of the University Hospitals of Cleveland, Ohio, USA (University Hospitals IRB #20210735). Semen and saliva samples were collected after written informed consent and were obtained by the Urology Department of the University Hospitals of Cleveland, Ohio, USA (University Hospitals IRB #20201481). Normal semen samples were purchased from Fairfax Cryobank (University Hospitals IRB #20201481) or obtained as part of a separate study (for samples with matched saliva) with California Cryobank/Cooper Surgical (University Hospitals IRB #20220558; NYU i19-00794). PBMCs were obtained from the Department of Otolaryngology of the University Hospitals of Cleveland, Ohio, USA (University Hospitals IRB #20191051), and from the Pediatric Hemato-Oncology Department of the Sheba Medical Center, Israel (Protocol #7079-09-SMC). University Hospitals, NYU, and Sheba Medical Center IRBs approved the study. All liver and kidney tissues were flash-frozen. Tissue sections were embedded in OCT and stained with hematoxylin and eosin. The slides were observed by the pathologist to ensure that no cancer cells were present in the tissues and evaluate for fibrosis. Male 8-week-old C57BL6/J mice were facilitated in the animal resource center at Case Western Reserve University of Cleveland, Ohio, USA (approved by Case Western Reserve University IRB #2020-0024).

### Data availability.

Sequencing data generated in this study have been deposited at the National Center for Biotechnology Information (NCBI) Database of Genotypes and Phenotypes (dbGaP) under accession code phs003476.v1.p1. The data are available under restricted access for genetic privacy and consent reasons, and access can be obtained by researchers for IRB-approved studies by application via the dbGaP website. Mouse sequencing data have been deposited at the NCBI Sequence Read Archive under accession code PRJNA1245429.

All code used in this study is publicly available.

Supporting data values for all figures are available in the supplemental Supporting Data Values file.

## Author contributions

The authors listed below have made substantial contributions to the intellectual content of the paper in the various sections described below: conception and design: SP, CAG, AT, RMS, BD, SR, MP, RL, AB, MGP, SM, PC, EL, ABS, RCO, CB, MG, BC, KD, LB, KVB, JH, QP, GDE, VAA, and JES; acquisition of data: SP, CAG, AT, RMS, SR, MP, RL, AB, MGP, ABS, RCO, MG, BC, KD, LB, KVB, QP, GDE, and JES; analysis and interpretation of data: SP, CAG, SR, RL, AB, MGP, SM, PC, EL, ABS, RCO, CB, KVB, QP, GDE, VAA, and JES; drafting of the manuscript: SP, CAG, AT, RMS, BD, SR, MP, RL, AB, SM, PC, EL, ABS, RCO, CB, MG, BC, KD, LB, KVB, JH QP, GDE, VAA, and JES; critical revision of the manuscript for important intellectual content: SP, CAG, AT, RMS, BD, SR, MP, RL, AB, SM, PC, EL, ABS, RCO, CB, MG, BC, KD, LB, KVB, JH, QP, GDE, VAA, and JES; statistical analysis: SP, SR, and JES; obtaining funding: JES; administrative, technical, or material support: RMS, SR, MP, RL, ABS, RCO, MG, BC, KD, AB, LB, KVB, QP, GDE, VAA, and JES; and supervision: JES.

## Supplementary Material

Supplemental data

Supplemental tables 1-8

Supporting data values

## Figures and Tables

**Table 1:**
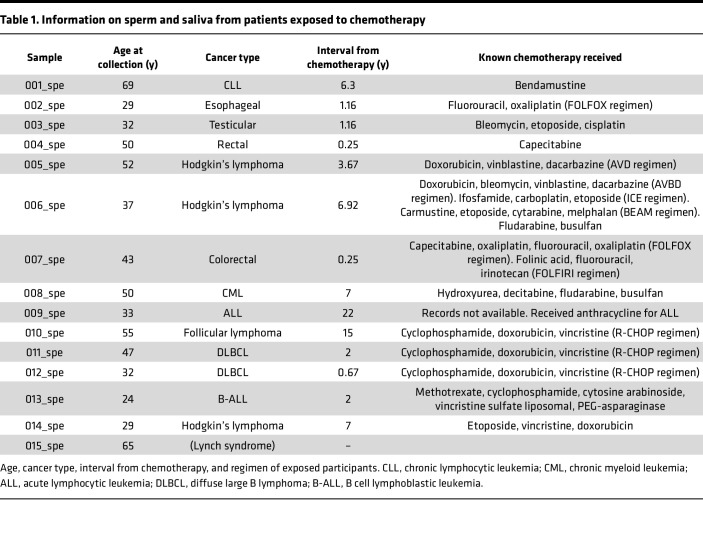
Information on sperm and saliva from patients exposed to chemotherapy

## Version 1 05/13/2025

In-Press Preview

## Version 2 06/23/2025

Electronic publication
