# Supplementary material for: Chemotherapy and the somatic mutation burden of sperm
Source: JCI Insight. 2025 May 13;10(12):e188175. doi: 10.1172/jci.insight.188175 (PMC12220962; doi:10.1172/jci.insight.188175)

Supplemental Figure S1. Assessment of somatic contamination in sperm.
A. Diagram of assay used to assess for somatic cell contamination. Four primer sets we used to detect somatic cell contamination in bisulfite treated DNA**. B-E**. Sensitivity of the indicated PCR reaction for somatic cell contamination using DNA from normal sperm and blood. Concentration of DNA and size measured using TapeStation. Of note, restricted DNA fragment represent sperm DNA whereas, non-restricted DNA fragments represent somatic cell DNA. For all reactions except PCR12 where restricted DNA fragments represent somatic cell DNA and non-restricted DNA fragments represents sperm DNA. **F-I.** Somatic cell DNA contamination test on chemotherapy treated sperm samples: Grid line represents concertation above which somatic contamination is detectable.

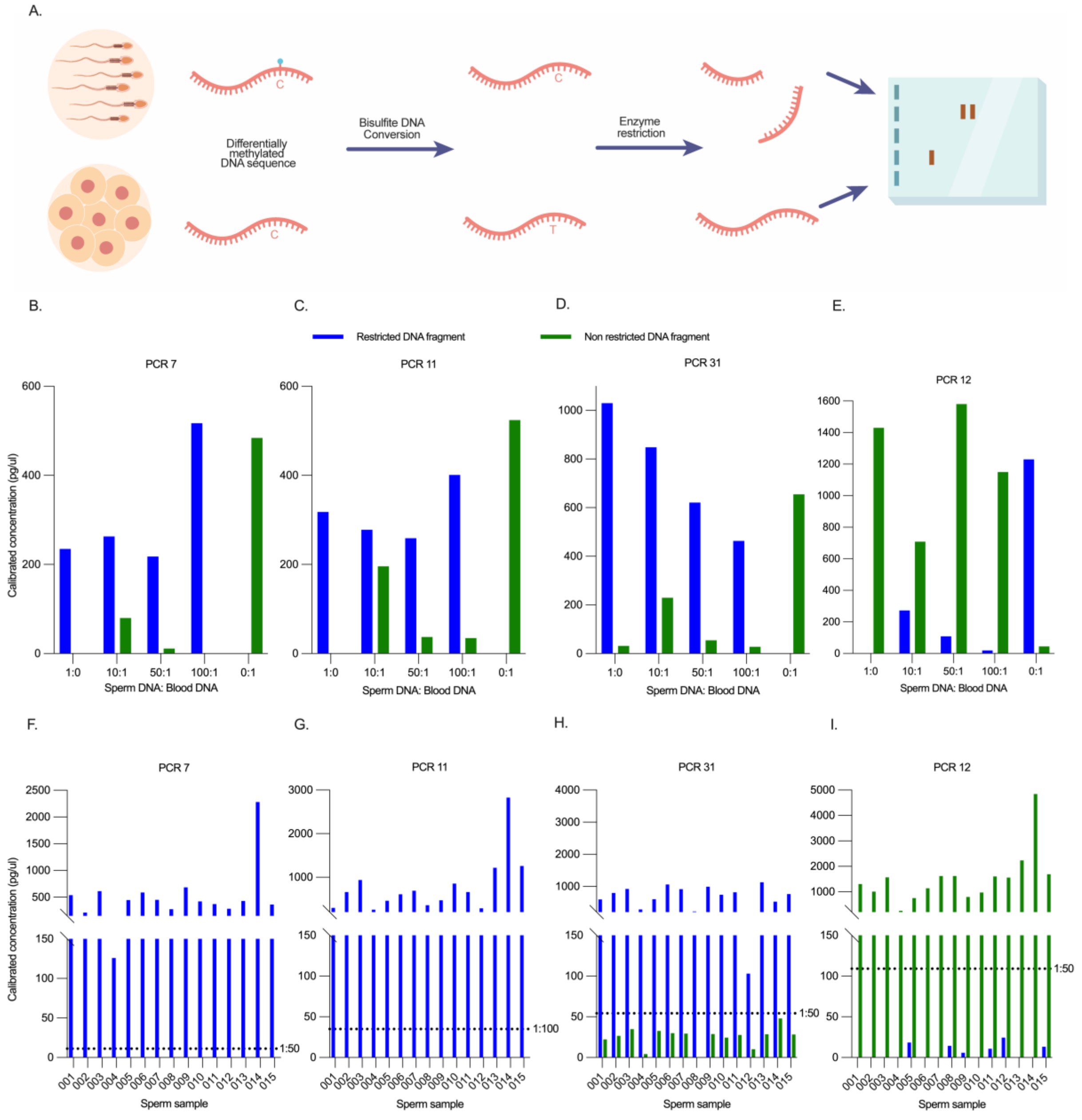

Supplemental Figure S2.
Mutational burden of sperm from subjects that received chemotherapy treatment compared to the interval from last chemotherapy treatment (R2=0.05250, simple linear regression analysis)

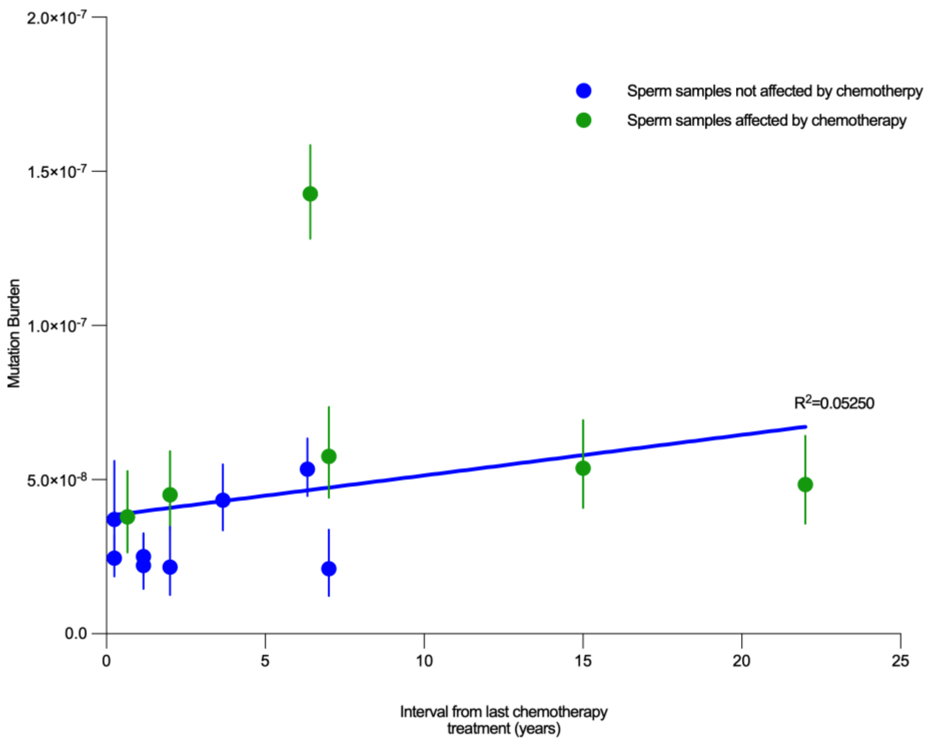

Supplemental Figure S3.

A. Data table of patients including: age, number of mutations detected in EcoSeq(39) **B**. Mutational burden for samples generated by EcoSeq(39) for cancer patients following chemotherapy treatment compared to subjects not exposed to chemotherapy. Figure includes our calculated Poisson 95% confidence interval for point estimates.

A.

Age at collection (years)	Number of detected mutations
6	11
8	4
8	14
16	9
18	16
6	6
8	7
15	9
16	6
19	13
6	4
7	2
11	1
11	4
13	2
13	4
14	3
15	5
15	3
18	8

B.

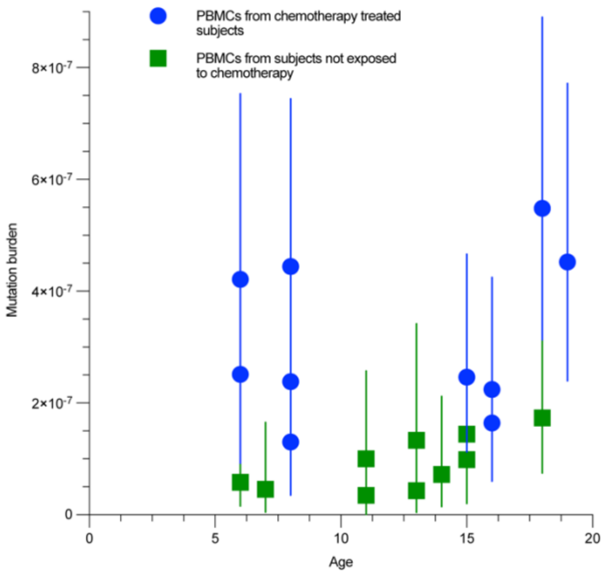

Supplemental Figure S4.
Mutational burden of liver tissue from patients not treated with chemotherapy sequenced by NanoSeq compared to previously published(7) data of estimated mutation rate in normal liver (P=0.1125, Y-intercept, simple linear regression analysis).

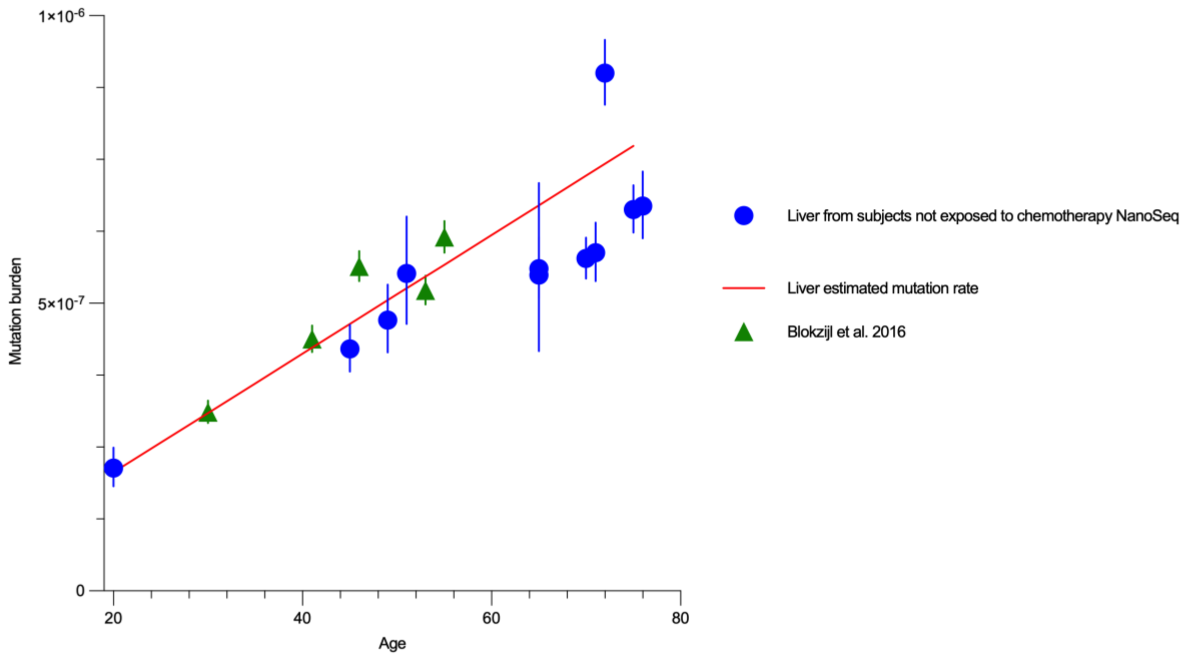

Supplemental Figure S5. A. Estimated contribution of select mutational signatures associated with aging (SBS1 and SBS5) and platinum chemotherapy (SBS31 and SBS35) across profiled specimens using a multiple linear regression model. **B.** Mutational signatures extracted and fitted using flexible Bayesian inference

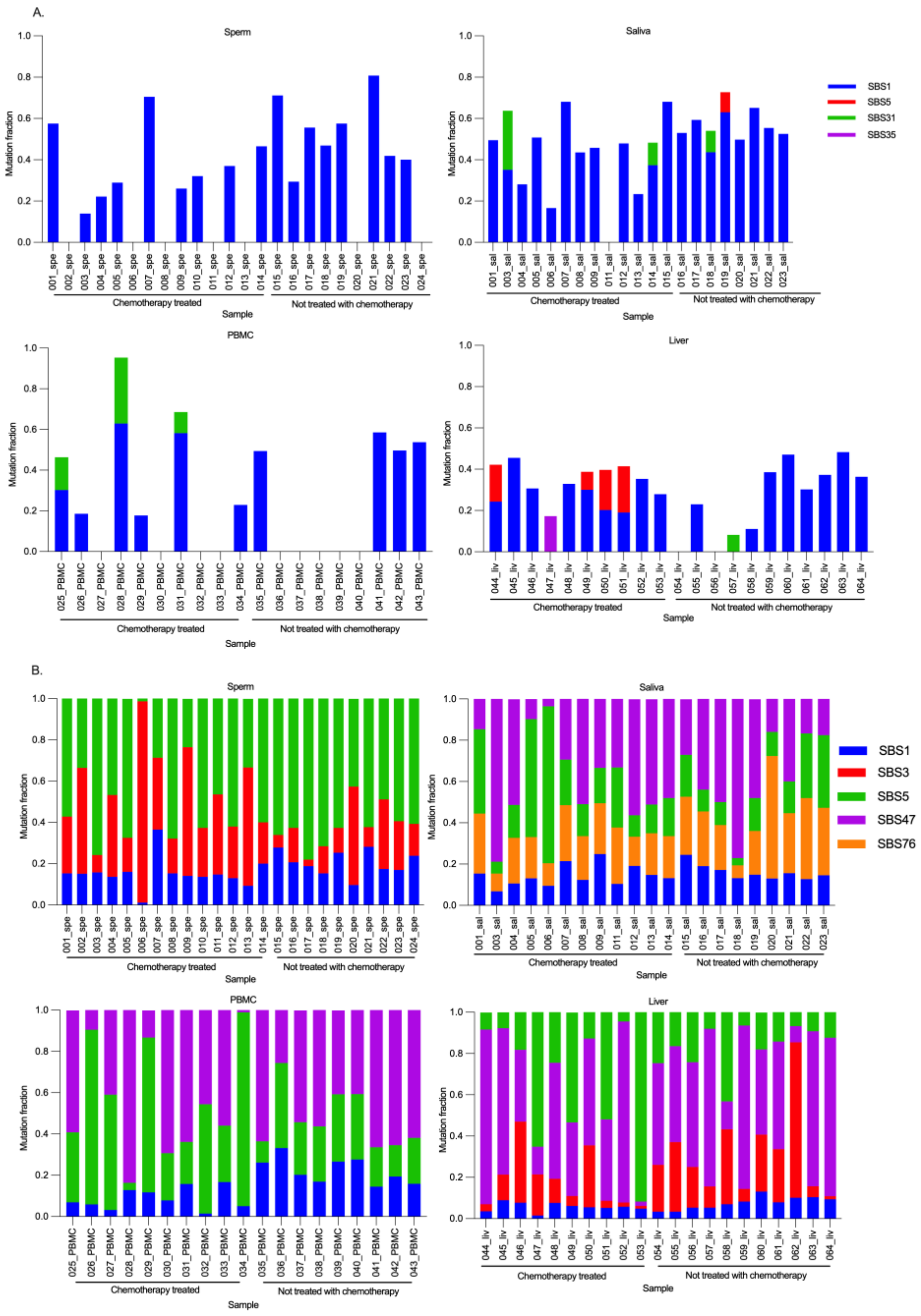

Supplemental Figure S6 A. Age, cancer type, interval from chemotherapy and regimen of exposed subjects **B**. Somatic mutation burden in kidney tissue from patients that received chemotherapy (right panel) compared to Kidney tissue from subjects not exposed to chemotherapy (left panel) **C.** Estimated contribution of select mutational signatures associated with aging (SBS1 and SBS5) and platinum chemotherapy (SBS31 and SBS35) across profiled specimens using a multiple linear regression model. **B.** Mutational signatures extracted and fitted using flexible Bayesian inference.

A.

Sample	Age at collection (years)	Cancer type	Interval from chemotherapy (years)	Known chemotherapy received
065_kid	48	Breast	6	Doxorubicin, Cyclophosphamide
066_kid	53	Multiple myeloma	1	Lenalidomide
067_kid	69	Breast	1	Doxorubicin, Cyclophosphamide

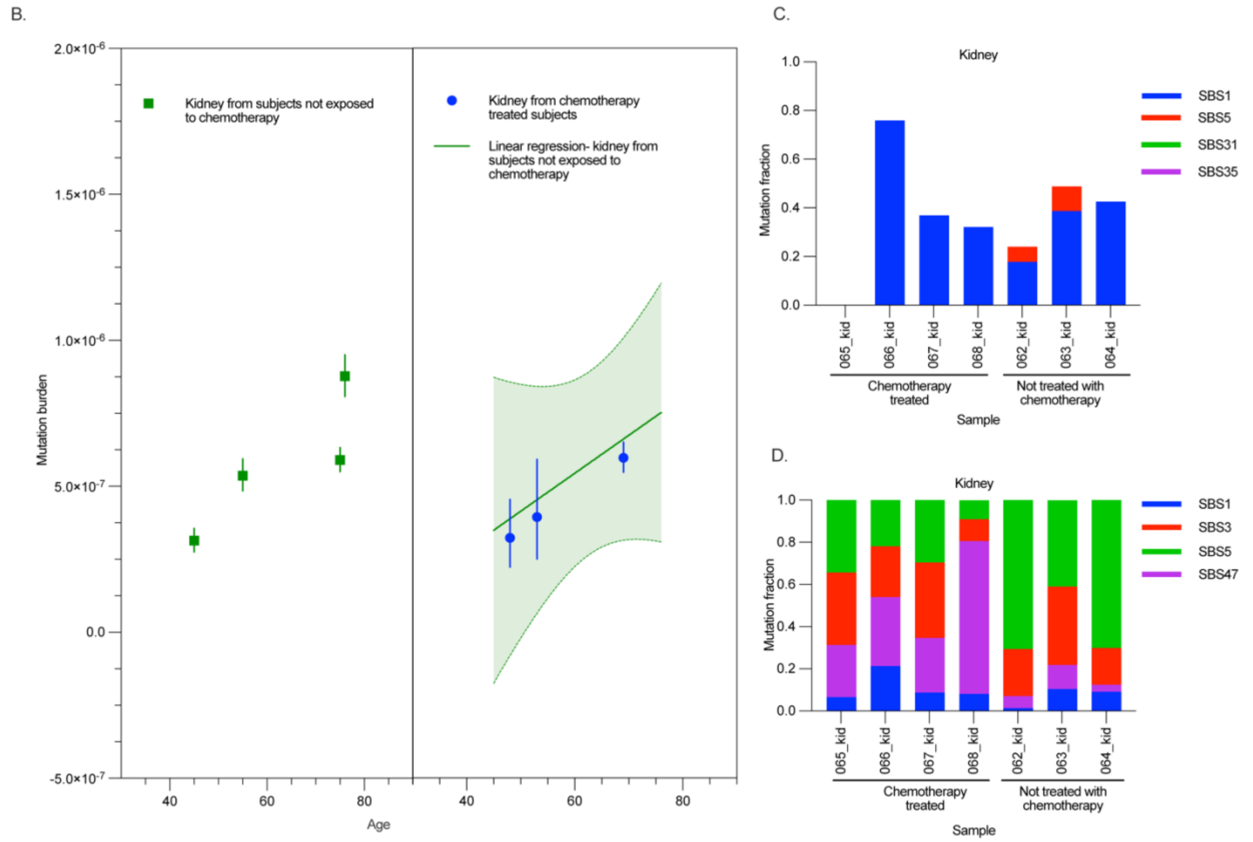

Supplementary Figure S7. Sperm from Human subjects.
Microphotography of sperm slides from participants after gradient centrifugation.

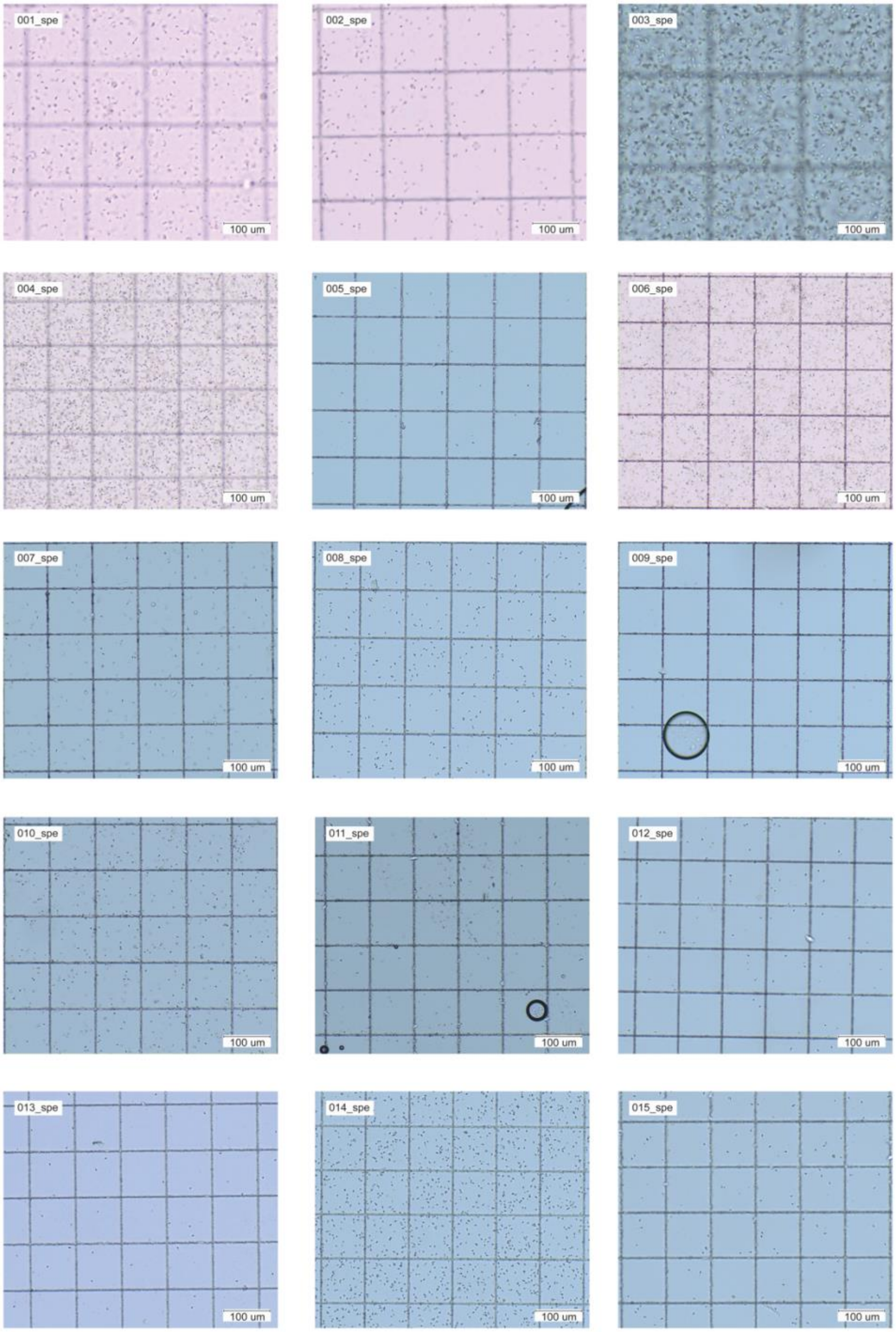

Supplementary Figure S8. Sperm from mouse subjects.
Microphotography of sperm slides from mouse subjects after gradient centrifugation.

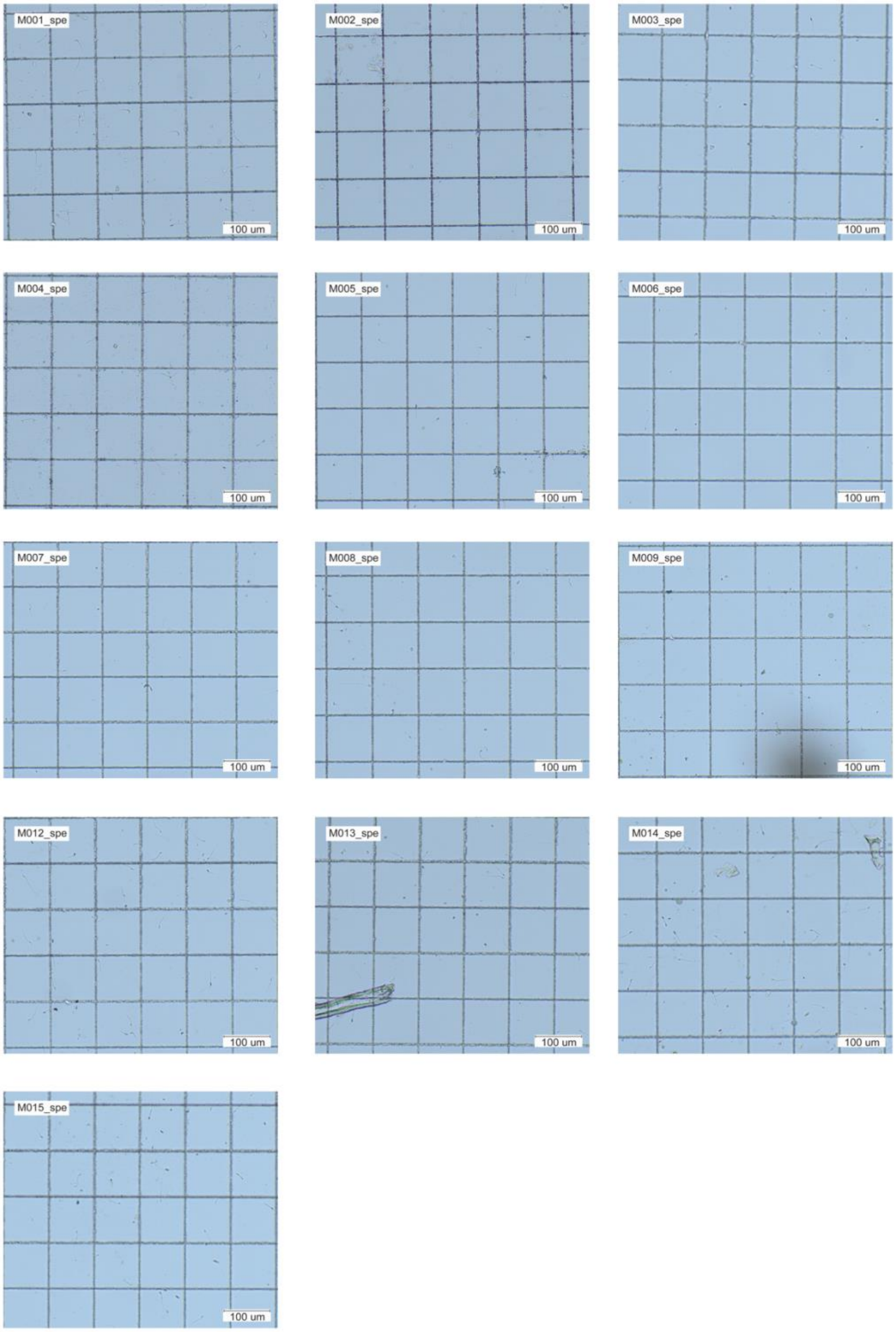

Supplement: Supplemental data [file jciinsight-10-188175-s177.pdf]
